# *Wolbachia* versus dengue: Evolutionary forecasts

**DOI:** 10.1093/emph/eot018

**Published:** 2013-09-11

**Authors:** James J. Bull, Michael Turelli

**Affiliations:** ^1^Department of Integrative Biology, The University of Texas at Austin, Austin, TX 78712, USA; ^2^Institute for Cellular and Molecular Biology, The University of Texas at Austin, Austin, TX 78712, USA; ^3^Center for Computational Biology and Bioinformatics, The University of Texas at Austin, Austin, TX 78712, USA; ^4^Department of Evolution and Ecology, University of California, Davis, CA 95616, USA; ^5^Center for Population Biology, University of California, Davis, CA 95616, USA

**Keywords:** intervention, biological control, cytoplasmic incompatibility, population suppression

## Abstract

A novel form of biological control is being applied to the dengue virus. The agent is the maternally transmitted bacterium *Wolbachia*, naturally absent from the main dengue vector, the mosquito *Aedes aegypti*. Three *Wolbachia*-based control strategies have been proposed. One is suppression of mosquito populations by large-scale releases of males incompatible with native females; this intervention requires ongoing releases. The other interventions transform wild mosquito populations with *Wolbachia* that spread via the frequency-dependent fitness advantage of *Wolbachia*-infected females; those interventions potentially require just a single, local release for area-wide disease control. One of these latter strategies uses *Wolbachia* that shortens mosquito life, indirectly preventing viral maturation/transmission. The other strategy uses *Wolbachia* that block viral transmission. All interventions can be undermined by viral, bacterial or mosquito evolution; viral virulence in humans may also evolve. We examine existing theory, experiments and comparative evidence to motivate predictions about evolutionary outcomes. (i) The life-shortening strategy seems the most likely to be thwarted by evolution. (ii) Mosquito suppression has a reasonable chance of working locally, at least in the short term, but long-term success over large areas is challenging. (iii) Dengue blocking faces strong selection for viral resistance but may well persist indefinitely at some level. Virulence evolution is not mathematically predictable, but comparative data provide no precedent for *Wolbachia* increasing dengue virulence. On balance, our analysis suggests that the considerable possible benefits of these technologies outweigh the known negatives, but the actual risk is largely unknown.

*Wolbachia* is a maternally transmitted bacterial symbiont of many insects [1, 2] and has several unusual properties that make it suitable for novel approaches to biological control of vector-borne diseases [3–5]. First, when introduced into an uninfected species, *Wolbachia* often increases the relative fitness of infected females so that the infection spreads to virtual fixation (hence it is frequently called ‘selfish’). Second, *Wolbachia* is compatible with a broad range of hosts, whereby a strain isolated from one species can often be successfully introduced to another species, even one distantly related [6–8]. Third, *Wolbachia* produces a range of effects that can be exploited for disease control: it can inhibit the growth of many other microbes in its hosts [9], it can shorten the life of its hosts [10, 11], and it can be used to potentially eliminate uninfected populations or populations whose *Wolbachia* is incompatible with the one being released [12, 13]. Despite these myriad effects, relatively little is known about the underlying mechanisms [14–16], in part because *Wolbachia* cannot be cultured *in vitro*.

The most ambitious application yet proposed for disease control using *Wolbachia* is dengue ‘elimination’. Dengue is a viral disease of humans, now endemic on three continents, affecting approximately one-third of the human population [17, 18]. The main vector of dengue virus (DENV) is the mosquito *Aedes aegypti*, which has no native *Wolbachia* infection. *Wolbachia* were introduced into *Ae. aegypti* with the hope of controlling DENV transmission by shortening the life of female mosquitoes [8]. Although life-shortening was projected to have only a small impact on mosquito demography, it could potentially have a major effect on disease transmission by greatly reducing the number of females old enough to transmit the virus [19, 20].

The life-shortening *Wolbachia*, denoted *w*MelPop, was initially found in a laboratory population of *Drosophila melanogaster* [21]. Initial tests of *w*MelPop in *Ae. aegypti* raised doubts about the feasibility of successful introductions in nature because *w*MelPop severely reduced both viability and fecundity [11, 22], making spread beyond isolated populations unlikely [23–25]. Fortuitously, it was discovered almost concurrently that some *Wolbachia* interfere with viruses and other microbes in the same host [26, 27]. The *Wolbachia* variant *w*Mel, originally found in natural *D. melanogaster* populations [28], partially blocks DENV transmission without greatly impacting *Ae. aegypti* [22, 29]. Field releases in isolated towns in northern Queensland, Australia, where DENV is not endemic but *Ae. aegypti* is, have successfully transformed the local *Ae. aegypti* populations, producing virtual fixation of DENV-blocking *Wolbachia*. Releases are underway to spread this infection in nearby urban areas, aiming for area-wide dengue control (S. L. O’Neill, personal communication).

All else equal, decreasing DENV transmission even slightly throughout an entire *Ae. aegypti* population might have a meaningful impact on human health due to the large number of infections [18, 30, 31]. Yet this optimism is justified only to the extent that evolution does not reverse the *Wolbachia* effect. What can we expect or *predict* about evolutionary responses to such a wide-scale intervention? Will dengue virus evolve to dodge the suppression? Will the virus evolve in ways that affect disease severity? How might *Wolbachia* be expected to evolve in this new host and how will the host evolve in response to this novel infection?

The choice of which *Wolbachia* strains to release is based on beneficial effects the strains exhibit at present. Yet these effects will likely change—evolve—after the release, and that evolution may alter the disease-control effectiveness of the released *Wolbachia* for decades to come. Successful *Wolbachia* introduction is nearly irreversible; additional *Wolbachia* infections might be used to displace the initial ones [4, 32, 33], but elimination of an infection, once established, is likely to be difficult. Hence, anticipating evolutionary changes of dengue–*Wolbachia*–mosquito interactions is important—and comparable to anticipating the evolution of resistance to pesticides and antibiotics.

## FRAMEWORK

Our goal is to suggest plausible paths of evolutionary change as it affects *Wolbachia*-based control of DENV: how likely is evolution to overturn an otherwise successful strategy? We also consider evolution of dengue virulence in response to these interventions. Beyond these, other possible effects of releases abound, spanning *Ae. aegypti* ecology [34], impacts on the larger insect community interacting with *Ae. aegypti* and impacts on non-dengue microbes found within this mosquito. We neglect these latter topics for lack of evidence on which to base predictions. Our primary concern is whether we can anticipate success or failure of attempted dengue reduction and whether dengue disease incidence and effects can be predicted to change. Furthermore, we consider only the evolutionary implications of these interventions, not practical, ethical, economic or efficacy issues related to them.

## THE BASES OF PREDICTION: NATURAL PATTERNS AND MODELS OF SELECTION

Evolutionary predictions can be founded on two types of evidence, and our predictions will rely on both. The most straightforward predictions are derived from actual observed evolution—experimental evolution or natural evolution (‘comparative’ evidence). In this case, the prediction is merely an extrapolation of evolution observed in one context to a new context. The second basis for prediction comes from models of natural selection, such as those that infer the fitnesses of alternative phenotypes in the context of specific ecologies [35]. Yet even when a phenotypic state has clear fitness benefits and the model has captured the relevant biology, evolutionary progress remains hostage to genetic variation. Thus, predictions of this second type require both an understanding of selection and knowledge of or assumptions about available genetic variation.

Predictions about evolution in this *Wolbachia*–DENV interaction are necessarily based on fragmentary evidence at this early state. Apart from simple cases like the evolution of insecticide resistance, evolution is often so sensitive to details that the only well-founded predictions are post hoc. If evolution in this system is highly sensitive to details—if our current predictions prove wrong or even prove right for the wrong reasons—our study will clarify the difficulty of making such predictions. The main hopes for successful prediction here, despite our ignorance of details, are ‘natural experiments’ that have been underway for decades if not millennia and interventions, analogous to pesticide applications, that impose such strong selection on the virus or *Wolbachia* that predictable evolution is expected except in the complete absence of relevant genetic variation. We will focus on these seemingly simple cases, offering predictions in advance of observed evolutionary outcomes.

## EXPECTED RESPONSES TO INTERVENTIONS

### Evolutionary responses to life-shortening: a clear expectation of reduced impact

Selection in response to a life-shortening maternal symbiont is aligned for both the symbiont and its host (Table 1). Under maternal transmission, *Wolbachia* should evolve to increase fitness of its female carriers [36]. On the basis of selection alone, therefore, we predict that *Wolbachia* strains that shorten host life will evolve to attenuate life shortening (as will their hosts), even though the efficacy of selection may be reduced at old ages [37]. Furthermore, any pleiotropic effects of these *Wolbachia* that manifest early in the mosquito life cycle will enhance this selection. This trajectory of reduced *Wolbachia* impact been observed in laboratory *D. simulans* transfected with *w*MelPop [38, 39]. Also, *Wolbachia* in natural populations of *D. simulans* have evolved over two decades to increase host fecundity [40]. Selection for *Wolbachia* to benefit their female carriers is also supported by other observations: defending hosts against other microbes and the obligate or near-obligate symbioses observed in many taxa (e.g. filarial nematodes [41], the parasitic wasp *Asobara tabida* [42] and various *Drosophila* [43, 44]).

**Table 1.** Life-shortening *Wolbachia*

Impact on dengue	Mosquito lifespan shortened so that DENV does not complete its life cycle, hence cannot be transmitted
Selection	*Wolbachia* and mosquitoes selected to extend female lifespan
	DENV selected for faster maturation
Genetic variation	*Wolbachia* strains vary in life-shortening effect, but variation within strains is unknown
	DENV can likely evolve faster maturation but with reduced transmission
Observed evolution	*Wolbachia* harm has evolved to reduced levels in caged and wild *Drosophila simulans*
Prediction	Life-shortening will attenuate in as little as a decade; while life-shortening persists, DENV will evolve faster maturation but with reduced transmission

Those direct observations from *Drosophila* suggest that a measurable reduction in life shortening may well occur in a decade or less. Reductions in life-shortening will enhance the spread of *Wolbachia* by lowering the unstable equilibrium frequency above which local infection frequencies tend to increase [23], facilitating both local introductions and spatial spread [24]. The negative effect on DENV transmission will be reduced and possibly eliminated as mosquito longevity recovers.

While host and *Wolbachia* are selected to attenuate life shortening, DENV would be selected to shorten its ‘extrinsic incubation period’ (EIP), the time it takes a mosquito that has just obtained a DENV-containing blood meal to be capable of DENV transmission [45]. There must be strong selection to shorten the EIP even in the absence of *Wolbachia*: daily survival rates in *Ae. aegypti* are on the order of 0.8–0.9, whereas females can typically transmit DENV only after 10 days or more ([8], cf. [20], but see [46] for short EIPs). Given that only a small fraction of mosquitoes live long enough to transmit DENV, selection on the virus to shorten its EIP must always be strong. The fact that a relatively long EIP persists in nature suggests either that a short EIP is impossible or entails a sharp decline in transmission rate; the latter alternative is supported by recent observations of short EIPs [46]. From these considerations, it seems that DENV could indeed evolve to decrease its EIP in response to life-shortening *Wolbachia*, but we infer that it would reduce its transmission rate to do so. A reduction in transmission should reduce disease incidence, but the magnitude of effect is difficult to predict.

A wild card in these forecasts is vertical transmission of DENV from the mosquito mother to her progeny. Vertical transmission would possibly allow a mosquito to transmit a virus acquired from her mother at an early age. Vertical transmission is apparently epidemiologically insignificant [47], but could evolve to higher levels under intervention. There are too many unknowns about such a process to make informed predictions, but the direction of evolution for DENV, *Wolbachia*, and the mosquito all coincide with intervention failure.

### Evolutionary responses to population suppression are less clear, but success is delicate

The expected evolutionary responses to *Wolbachia*-based population suppression are less straightforward (Table 2). In both naturally infected and transinfected mosquitoes, matings between *Wolbachia*-infected males and uninfected females (or females carrying an incompatible *Wolbachia* variant) produce embryo mortality at or near 100%. This ‘cytoplasmic incompatibility’ (CI) was first identified in the mosquito *Culex pipiens* [48], and Laven [12] demonstrated that releasing incompatible males could eradicate an isolated disease-vector population of *Culex pipiens fatigans*. This approach is functionally analogous to the release of radiation-induced sterile males, which has proven effective against some but not all pest species [4, 5, 49].

**Table 2.** Population suppression

Impact on dengue	Mosquitoes eliminated or reduced in number
Selection	Female mosquito strongly favored to survive *Wolbachia* killing or avoid mating with *Wolbachia*-bearing males
Genetic variation	No apparent standing variation for CI resistance in *D. simulans*
Observed evolution	Wild *Drosophila* have not evolved to suppress incompatibility in the short term but have in the long term; *Hypolimnas bolina* evolved to resist male killing within a century, whereas *D. innubila* has not
Prediction	Population suppression will likely remain effective over a decade or more; long-term success will be diminished by the combination of accidental releases of females from the suppressing strain, paternal transmission of *Wolbachia*, and evolution of mating discrimination. Economics of continual release required for long-term suppression will limit applications of this technology

In any regime that kills entire populations, there is intense selection for escape—as learned countless times from resistance evolution to pesticides and antibiotics. The speed of local population collapse under massive male releases is such that little if any gradual evolution of escape is expected, but escape can emerge in other ways. First, any existing mutants capable of surviving the cytoplasmic incompatibility will be favored outright. Second, if the sterile male release is not large enough to extinguish the local population, or the population extends beyond the release site, female mating discrimination can evolve gradually in zones of partial suppression [50]; if practical, local genetic variation from the wild strains could be introduced into the captive stocks to mitigate discrimination. Third, any paternal transmission of *Wolbachia* to viable progeny or accidental release of the *Wolbachia*-bearing females from the suppressor strain [4] will create a wild mosquito strain no longer suppressed by that *Wolbachia* (in this sense, *Wolbachia*-induced sterility differs fundamentally from the irradiated sterile male technique). Regardless of escape mechanism, it is easily appreciated that the attempt to suppress a large mosquito population will face greater difficulties than attempts to suppress small ones, and long-term suppression will be more challenging than short-term suppression.

In many of these scenarios, the outcome rests on the existence of appropriate genetic variation. The comparative data, considered next, provide a mixed message. Over evolutionary time scales, hosts have evolved to suppress *Wolbachia*-induced mortality. During the 20th century, the moth *Hypolimnas bolina* evolved to suppress male killing by *Wolbachia* [51, 52]. Similarly, both comparative and experimental evidence suggest that *D. melanogaster* has evolved to suppress CI [53], but the age of this *Wolbachia*–host association is on the order of 8000 years [54].

In contrast, despite constant selection associated with the persistence of uninfected individuals produced by imperfect *Wolbachia* transmission, *D. simulans* in California has not evolved to suppress CI over the past 20 years (about 200 generations; [55]). Moreover, *D. innubila* has not evolved to suppress *Wolbachia*-induced male killing over many thousands of generations [56]. Hence, over the time scale of a population suppression effort, there might well be no significant evolution in mosquitoes to escape.

### Blocking DENV: partial success expected

Some strains of *Wolbachia* appear to block DENV transmission. At face value, introduction and spread of those strains offers the hope of a profound suppression of DENV incidence without changing mosquito demography.

#### Selection

The ramifications of and expected evolution in response to *Wolbachia* that block DENV are more complicated than in either of the previous two cases (Table 3). Selection on the virus is straightforward: there is strong selection for viruses to avoid blocking. Direct selection on *Wolbachia* to block DENV is weak or absent.

**Table 3.** Dengue blocking

Impact on dengue	Virus infects but cannot disseminate from mosquito
Selection	Strongly asymmetric: DENV strongly favored to escape, *Wolbachia* not obviously selected (directly) to maintain blocking
Genetic variation	Unknown
Observed evolution	*Wolbachia*-infected mosquitoes transmit many human viruses but are commonly associated with reduced viral transmission
Prediction	DENV will evolve to reduce complete blocking by *Wolbachia*, but partial DENV blocking will persist indefinitely

The latter conclusion requires elaboration. Selection should certainly favor or reinforce blocking to the extent that viral infection reduces mosquito female fitness (as found for DENV-2 by [57]). But for blocking to be favored, blocking must restore female fitness. It is not immediately obvious that virus blocking per se benefits *Wolbachia*, as the virus is observed to replicate in some tissues of the mosquito even when transmission is blocked [58]. Furthermore, the magnitude of selection on *Wolbachia* is only as strong as the net effect of DENV on mosquito/*Wolbachia* fitness. If DENV infection frequencies in mosquitoes are on the order of 1% [59] and the fitness reduction associated with infection is on the order of a few percent [57], selection on *Wolbachia* to protect its mosquito host from DENV is weak at best.

If direct selection on *Wolbachia* for blocking is weak, indirect selection could be important. For example, blocking DENV may be a simple mechanical consequence of *Wolbachia* filling salivary gland cells and physically limiting resources for the virus [60]. Thus, direct selection for high *Wolbachia* somatic density may indirectly select for blocking. Conversely, selection may be in the other direction: lower somatic densities are found with more beneficial *Wolbachia* and seem to follow recent transfections [38]. Furthermore, there can be a significant deleterious fitness effect of *Wolbachia* in a new host (see also [22], on the order of 10% in field data with wMel [29]). Thus, evolution of reduced blocking could be rapid following an introduction. Mechanisms of dengue-blocking and host fitness reduction remain speculative [61], however, and are vital for understanding *Wolbachia*’s pleiotropic effects and their ramifications for evolution.

Analyses of *Wolbachia*-infected *Ae. aegypti* established in small Australian towns for 2 years indicate no significant attenuation of virus blocking (Frentiu *et al.*, submitted for publication). Those towns lacked endemic DENV, so any possible viral adaptation could not be assessed. However, the persistence of DENV blocking in these populations—and of virus blocking in natural populations of *D. melanogaster* in which *Wolbachia* does not cause CI—suggests that virus blocking does not require deleterious effects on the insect host.

Despite uncertainties about the bases of DENV blocking, the strong asymmetry in selection on DENV versus *Wolbachia* supports a prediction of viral evolutionary superiority. As will be argued next from direct observations, there appears to be a limit to that superiority. Countless examples of viral escape from human interventions likewise favor the verdict of viral supremacy in this case. Yet viral escape from *Wolbachia* blocking is not assured. Despite a near ubiquity of viral escape from single drugs, the simultaneous use of three anti-HIV drugs (known as HAART) seems sufficient to contain HIV evolution of resistance within patients. Some viral vaccines have been used globally for half a century without any noticeable viral escape (e.g. polio, measles). The critical determinant may be the ‘dimensionality’ of the challenge to the virus—how many mutations are required simultaneously to overcome the barrier. The exact mechanism of *Wolbachia* blocking of dengue is unknown, but it seems to be multifarious [61, 62], so the blocking could involve a multidimensional challenge to the virus. The comparative data on DENV transmission by *Ae. albopictus*, which is naturally infected with *Wolbachia*, indicates that viral escape from transfected *Ae. aegypti* is far from certain, as considered next.

#### Comparative evidence

The evolutionary fate of the *Wolbachia*–dengue interaction in *Ae. aegypti* might be inferred from naturally occurring *Wolbachia*–virus interactions: is *Wolbachia* infection of a mosquito commonly associated with inability to transmit arboviruses? The answer is clearly no with respect to complete blocking. *Culex pipiens* and *Aedes albopictus* are common mosquito species that harbor *Wolbachia* [63–65]. Both species are vectors for many arboviruses (as listed on the CDC arbocat site at http://wwwn.cdc.gov/arbocat/). Indeed, *Ae. albopictus* transmits DENV and has caused dengue epidemics [47]. *Aedes albopictus*, with its *Wolbachia* infections, is also a major vector of chikungunya virus. Studies of chikungunya virus dynamics in *Ae albopictus* reveal a decline in *Wolbachia* density as the virus life cycle enters the transmission stage [66], as if the virus is reversing interference by *Wolbachia*. None of this points toward *Wolbachia* supremacy.

One limitation of these comparative data is that they are one-sided—the fact that a mosquito harboring *Wolbachia* transmits some viruses but not others could indeed reflect blocking of the missing viruses. Blocking cannot be inferred without direct experiments, whereas the absence of blocking is self-evident for the transmitted viruses. A second limitation is that the data are qualitative, not quantitative. In particular, various data indicate that ‘native’ *Wolbachia* infections reduce arbovirus transmission even though they do not completely block it. Transmission rates of West Nile virus by *Culex quinquefasciatus* are reduced 2- to 3-fold by the native *Wolbachia* [67]. *In vitro* assays of *Ae. albopictus* transmission suggest that the native *Wolbachia* completely blocks DENV-2 transmission [58]. These laboratory data corroborate meta-analyses that *Ae. albopictus* has significantly lower vector-competence for DENV than *Ae. aegypti* [47]. The latter study also reviewed ‘natural experiments’ indicating that, on islands such as Taiwan, Guam and Hawaii where *Ae. albopictus* has become the dominant dengue vector, dengue epidemics are much less frequent and less severe than on comparable islands with *Ae. aegypti* transmission.

Overall, the comparative evidence offers encouragement that *Wolbachia* may provide lasting, quantitative reduction in transmission of some DENV serotypes. At the same time, it seems likely that any such blocking will not fully avoid viral escape and may even vary with the mosquito genotype, as does vector competence [68]. A quantitative reduction in transmission can lead to meaningful reductions in numbers of cases, so implementation of the *Wolbachia* strategy should not rest on complete blocking (see [30] as an encouraging example, but [31] as an indication of the complexities in making robust predictions about the impact of reduced transmission on disease prevalence).

## EVOLUTION OF DENGUE VIRULENCE IN RESPONSE TO *WOLBACHIA*: NO PREDICTION

Theoretical considerations have revealed that parasite virulence can evolve in response to many interventions, including vaccines [69–71]. *Wolbachia* can alter both the viral life history in the mosquito and the mosquito life history, and both can theoretically affect evolution of viral virulence in humans and mosquitoes. Might *Wolbachia* select a nastier strain of DENV? Can we make an informed prediction about evolution of DENV virulence in response to *Wolbachia*?

### Inference from models

The short answer is that current evolution-of-virulence models cannot be relied on to confidently predict changes in dengue virulence. As background, it is important to understand that virtually all evolution-of-virulence models assume a genetic ‘trade off’ between viral transmission and virulence; virulence in turn is taken as host death rate. Most models address the equilibrium virulence, the state of virulence when no further evolution is favored by natural selection, and they compare the equilibrium level of virulence expected under alternative scenarios (e.g. with and without intervention). Furthermore, evolution-of-virulence models typically neglect the many environmental variables that can have profound effects on disease severity and can even alter the course of virulence evolution (e.g. [72]).

A basic issue when applying standard evolution of virulence models to DENV-blocking *Wolbachia* is whether observed dengue virulence actually corresponds to an equilibrium state as envisioned by the models. The standard model used to study virulence evolution is a ‘SIR’ model that counts susceptible hosts (S), infected hosts (I) and recovered hosts (R). A quantity critical to understanding virulence evolution is the number of transmissions over the lifetime of an infected host, which is found as the ratio (transmission rate)/(host death rate + recovery rate).

A variant with higher virulence is expected to be favored if it increases this ratio—if the increased death rate it causes is more than offset by its higher transmission rate. The tradeoff dictates that it cannot increase transmission rate without also increasing death rate.

Applying this model to current dengue virulence in the absence of *Wolbachia* interference, one would expect a high enough human death rate per infection to limit DENV transmission from the host. Although accurate numbers are difficult to obtain, the mortality rate over all dengue infections appears to be on the order of 0.001 or less ([17, 73]; http://www.who.int/csr/disease/dengue/impact/en/); the case fatality rate can vary several-fold over the course of an epidemic but is still low [74]. Furthermore, recent work suggests that the estimated number of dengue infections is possibly 4-fold times the apparent infection rate because of asymptomatic infections [18], further depressing the case mortality rate. As the recovery rate from DENV infections is high and the death rate very low, the ratio for DENV lifetime transmissions is insensitive to increases in death rate. It follows that increases in transmission should be favored unless they increase host mortality profoundly—i.e. unless there is an extraordinarily steep tradeoff. Such steep tradeoffs are unknown, raising doubts about the applicability of this type of model to explain current DENV virulence as an evolutionary equilibrium.

The problem of predicting DENV virulence evolution goes further. A recent model of virulence evolution of an arbovirus required parameterization of four tradeoffs affecting virulence in humans [75]. One of these tradeoffs is supported [76]: viral titer in humans correlates positively with transmission to mosquitoes (parameter *β* in their model); but that leaves three other tradeoffs unanswered. As the authors emphasized, an intervention such as dengue-blocking *Wolbachia* may either favor an increase or decrease in virulence depending on these unknown constraints.

### Comparative evidence

If *Wolbachia* blocking generally selects increased virulence of arboviruses, one might expect higher virulence in viruses transmitted from mosquitoes infected with *Wolbachia*. As noted above, *Wolbachia* infections of *Ae. albopictus* partially block DENV transmission. For at least several decades, *Ae. albopictus* has been the dominant dengue vector on several Pacific islands and in areas of southern Asia. As noted by Lambrechts *et al.* [47], dengue epidemics seem systematically less severe in these areas than in comparable locales in which *Ae. aegypti* is the dominant vector. Furthermore, there is no suggestion that DENV has become more virulent where *Ae. albopictus* is the dominant vector [47]. The limited comparative evidence thus goes against evolution of higher DENV virulence in response to *Wolbachia* blocking. As pointed out by a reviewer, evolution of virulence is a minor consideration in areas with short-lived epidemics of DENV, where the virus dies out between successive introductions.

## DISCUSSION

A radical effort is underway to limit and possibly eradicate dengue virus, an arbovirus transmitted among humans by mosquitoes of the genus *Aedes*. In contrast to the standard approaches of environmental dosing with chemicals, repeated introductions of short-lived biological agents, or a vaccine, population transformation with *Wolbachia* aims at long-term biological control. Local introductions of *Wolbachia* have the potential to spread widely and ultimately thwart the mosquito’s ability to transmit the virus by either of two mechanisms, depending on strain: (i) direct blocking of transmission or (ii) shortening mosquito lifespan so that she cannot mature the viral infection. Additionally, *Wolbachia*’s property of cytoplasmic incompatibility in crosses of infected males with uninfected females enables a third intervention, (iii) depressing local mosquito populations by releasing ‘sterile’ males. However, this latter method requires the continual release of lab-reared strains and thus depends on a substantial infrastructure (e.g. [13]).

The first two methods are unusual forms of biological control, because instead of killing the target species, *Wolbachia* merely spreads in the mosquito population and blocks DENV transmission. As *Wolbachia* derives no obvious benefit from reducing the mosquito’s ability to transmit the virus, the question is whether the release of a *Wolbachia* strain that currently reduces dengue transmission will persist in this effect. Our focus is reviewing the bases for predicting alternative outcomes.

The following summarizes our conclusions.

1. *Evolution of reduced harm by* Wolbachia. In many interactions, *Wolbachia* and host evolve toward mutualism. Use of a life-shortening *Wolbachia* to kill mosquitoes before they can transmit DENV is thus likely to provide at most only a short-lived benefit. Because substantial fitness costs increase the threshold frequency that must be surpassed for *Wolbachia* to spread, life-shortening *Wolbachia* will be relatively difficult to establish and are likely to spread slowly if at all [24, 25]. Introducing one *Wolbachia* strain may interfere with the subsequent introduction of other strains later found to have more desirable qualities, so introductions should be limited to those bacteria with a high probability of success.
2. *Mosquito evolution in response to CI*. Mating with *Wolbachia*-infected males can effectively sterilize uninfected/incompatible females, and infected-male releases can be used to suppress mosquito populations [12, 13]. Success can be undermined by evolution in the target mosquito population to suppress CI, but various lines of evidence suggest that genetic variation for resistance to CI is sometimes absent. Alternatively, the strategy runs a risk of failure from even rare paternal transmission of *Wolbachia* into the target species or from accidental release of female mosquitoes bearing the *Wolbachia* strain. Short-term success is thus feasible, but long-term success faces several challenges. Some mechanisms of failure can be overcome by introducing new strains of *Wolbachia* into the mosquitoes used for suppression.
3. *Viral escape from transmission block*. Some *Wolbachia* block DENV transmission. The release of such a strain thus offers the possibility of DENV eradication, if the blocking is not overcome by viral or bacterial evolution and is invariant across the mosquito population. The comparative evidence shows that some strains of *Wolbachia* allow viral transmission by mosquitoes, raising the possibility that evolution may ultimately reverse blocking. Combined with the strong asymmetry in selection on virus versus bacterium, the expectation is that dengue will evolve to overcome an absolute block to transmission. The time course is difficult to predict from comparative data but could be on the order of a decade or less because of the rapid evolutionary potential of the virus. Yet comparative evidence suggests that at least partial blocking will persist long term, and the long-term persistence of complete blocking is not out of the question. The quantitative impact of partial blocking on disease incidence is difficult to predict but could be meaningful because so many humans are at risk and dengue transmission rates tend to be relatively low [31].
4. *Changes in virulence*. A concern is that a successful *Wolbachia* intervention may select higher virulence in DENV. At this stage, however evolution of dengue virulence cannot be predicted even qualitatively. From comparative data, no unusually lethal viruses have been tied to *Wolbachia* presence in other vectors, and epidemics vectored by *Aedes albopictus* (which harbors *Wolbachia*) are noted to be less severe than those from *Ae. aegypti*. Although there is no basis for predicting the evolution of higher DENV virulence in response to virus blocking by *Wolbachia*, there is likewise no sound basis for rejecting the possibility of higher virulence evolution – neither null model can be rejected.

As with most biological control agents, introduction of *Wolbachia* into a wild population is essentially irreversible: the bacterium is likely to remain with the host indefinitely (unless replaced by another *Wolbachia*). However, other *Wolbachia* strains can be introduced on top of existing strains, with double infections (or incompatible infections) replacing the single infections (e.g. [77]). Alternatively, captive populations of the host can be cured of their *Wolbachia* and infected with other strains [4]. Release of sufficient numbers of hosts infected with another strain can cause displacement of the original strain if the two strains are incompatible [24, 32]. Some species exhibit a bewildering array of *Wolbachia* strains, and we do not yet understand the complexities of coexistence [78], so our views of strain replacement are undoubtedly naïve.

The *Wolbachia* releases underway provide a novel opportunity to make a priori predictions about many aspects of the near-term and long-term evolution of a selfish bacterium, a virus, and the insect host/vector. No doubt many similar opportunities for prediction will be soon afforded by the release of genetically modified organisms on what we imagine will become a vast scale. There is considerable uncertainty in anticipating some evolutionary consequences of *Wolbachia* on dengue, but we can marshal some evidence to identify likely outcomes, such as dengue virus partially escaping transmission blockage and *Wolbachia* quickly reducing its deleterious effects on the mosquito. These predictions may fail, of course. But having offered them in advance should help refine future prediction.
